# Epidemics and Their Every Day Causes

**Published:** 1858-10

**Authors:** W. I. Cox


### ORIGINAL COMMUNICATIONS.

### EPIDEMICS AND THEIR EVERY DAY CAUSES.

By W. I. COX, M.R.C.S.

[*Concluded from p. 70.*]

### III.—THE USE OF FOUL WATER.

It is with considerable diffidence that I venture to add my mite of observation on this head, to the gigantic labours of one of the most talented and indefatigable philosophers of the day—Dr. Snow. Foul sources of water-supply form a very common and little regarded evil appertaining to dwellings otherwise well appointed and salubrious. How frequently in country villages may be seen a pump, or draw well, in the midst of a farmyard or filthy court; receiving the surface drainage of heaps of stable manure, pigsties, etc. What is the almost inevitable result?—Water highly charged with organic matter in a decomposing state gradually, but constantly, percolates through the shallow stratum of soil, and effectually contaminates the source where the house supply is obtained. How often do we notice green, slimy, stagnant pools, in the close vicinity, and affording the sole water supply of cottages. It is unquestionable, that one of the most frequently occurring causes of impurity, is the contamination of wells and streams by sewage and percolation through soils saturated with organic fæcal matter. Of course, *slow* percolation through a deep and gravelly soil would effectually rid the surface water of its organic impurity. But this is not often the case. It is well known that surface water possesses a strong affinity for organic matters, which are diffused over every surface in the neighbourhood of living beings. The large relative amount of the *nitrates,* found in even very deep wells in large towns and near graveyards, sufficiently testifies to this fact.

Waters tainted with various organic matters,—whether gaseous, as carbide or sulphide of hydrogen; or solid, as putrescent vegetable fibre; or vitalized, as algæ, confervæ, hydræ, fungi, infusoria, etc.,—are a very frequent cause of severe visitations of *bowel complaints* during the summer months. Several instances came under my own observation in 1853 and 1854, of the aggravation of *epidemic diarrhœa* from this cause. That water falling on a growing soil, and running off to lie in stagnant pools, is sure to become tainted with animal and vegetable life, is well known; and when to this is superadded the circumstance of the said soil being highly charged with effete organic products, the water thus collected must necessarily be highly impure, and most unfit for human consumption. Yet very often it forms the only available source of supply. As an instance, I may mention a large piece of common land in the districts wherein I now reside, on which stand thirty or forty cottages; the inhabitants of which use no other water than that afforded by surface pools; the majority of which are, during the summer and autumn, covered with vegetable life, slimy, and bubbling with the liberated products of decomposition. They are all uncovered, sunk below the general level of the ground. Hundreds of donkeys, pigs, and geese, graze close to the brink. The population in these cottages are of stunted and miserable appearance. Several deaths occurred among them from choleraic diarrhœa last autumn: the neighbouring village remaining free from such a visitation. Whenever fever occurs there, it is always of bad type and difficult to eradicate. I have examined the water from the largest pool; it contained (September last) five grains of organic matter in the imperial gallon.*

*Scarlatina simplex* was epidemic in a small agricultural village, in the west of England, in August 1856. There occurred in all, thirty-eight cases, chiefly among the peasantry; whereof three proved fatal. Now *two* of these fatal cases were in *one* house, the residence of a wealthy farmer. The disease therein changed its character; assuming the worst asthenic type, with intense throat affection, and, as is so frequently the case, defying all treatment. The persons attacked were a servant girl and three children. The two eldest children, aged respectively seven and five years, died. The younger child and servant recovered with some difficulty. In no other house in the village, not even in the poorest cottage, did the disease take on the malignant type. This sad visitation was considered mysterious and wholly unaccountable. The following facts, however, seem in my humble judgment to elucidate it. The water supply of the farmer's family I found to be derived from a shallow drawwell in the back yard, imperfectly covered; surrounded by heaps of decomposing manure and cowsheds, —the black drainings from which were constantly flowing over the soil. I examined the water from this well on two occasions, *before* and *after* heavy rains. The first analysis showed sixty grains of solid matter (chiefly nitrates) in the gallon; whereof five grains were undecomposed organic matter. The

* Water containing more than four grains in the gallon is unfit for use.

second analysis (after the rain) gave the enormous amount of between seven and eight grains of organic matter. On the latter occasion, the water in large bulk showed a greenish tinge of colour; and exhaled a disagreeable odour after having been kept in a closed vessel for ten days. The rest of the village derived its chief supply of water from a good public well, situated at a little distance in a large field, and well covered from the weather.

The dependance of outbreaks of *cholera* on the use of foul water, is now too well known to be questioned, even by the most sceptical on such points.

In a horrible plague spot near the metropolis, which existed until very lately in Kensington parish, there was afforded, in September 1849, a practical example of the fatal effects of the accumulation of filth around dwellings, where the water is not brought from a distance; and of the absurdity of hoping that such consequences can be confined to the neighbourhood where they originate. This place (called the Potteries), comprised about two hundred and forty houses, inhabited by eight hundred and sixty wretched beings; as filthy and degraded as in the south of Ireland. The place also abounded in pigsties. There was no sewerage,—no drainage. Water was properly supplied only to sixty houses The remainder derived their supply from foul ditches, stagnant fetid pools, and a few shallow wells on the premises; the water of which latter (from the organic matters soaking into them), was equally noxious with that of the pools and ponds. I examined four specimens of the water used by these people,—two samples from wells, and the remainder from the largest of the pools above-mentioned.

No. 1, from the deepest of the wells, contained fifty grains of solid matter in the gallon, chiefly the nitrate salts. No. 2, fifty-six; six grains of which were organic matter. The other two specimens yielded seven and eight and a-half grains respectively, in the gallon, of organic matters. These last specimens were foul in appearance, and exhaled an offensive odour. Asiatic cholera broke out in this locality with dreadful virulence in the month above-named; carrying off from the slender population two and three a day. It speedily reached a new and well built terrace of good houses, situated at the distance of a quarter of a mile; whence it swept away eight lives in the course of six days.* In 1853, another outbreak occurred in this place, attributable to the same cause.

In September 1853, the cholera suddenly appeared in the

---

* See the first volume of "Household Words", p. 463.

district of Kensal New Town, where I was then resident. Seven deaths occurred during the first week of its visitation. With one exception, however,—the case of an habitual drunkard,—its victims were *exclusively* confined to those of the inhabitants whose dwellings had *no water* "laid on," and who used for household purposes the abominable contents of the canal, or "cut", bordering the district. On examination, I and other observers found this water highly charged with organic matters. How, indeed, could it be otherwise? Chamber utensils were being constantly emptied into it at all points; whilst the carcases of dogs and cats and animal refuse of every description were frequently to be seen floating on its surface.

The town of Bridgend, in Glamorganshire, is healthily situated; but, until very recently, its water supply was dependent on the river on the banks of which it is built. As this stream (a mere rivulet in summer) received all the sewage, and human egesta were poured into it from a hundred houses, its waters must necessarily have been, especially in the season of drought, very unfit for use in the lower parts of the town. For many years past it was remarked, that fever visiting this locality, generally assumed *a low and fatal type*. Cholera, however, was a stranger to it until, in August 1854, this dreaded visitant appeared suddenly, whence, no one could say; and carried off several of the inhabitants of the lower portion, in a few days.

### IV.—PRIVATION OF SOLAR LIGHT.

Light is of very great importance to the health and well-being of the human race. Self-evident and trite as this statement may appear to be, its practical importance is only just beginning to be felt, even in this highly civilized and scientific age. To healthy life of body and mind, the luminous principle of the sunbeam is certainly essential. This truth, however, has been disregarded to a melancholy extent in the construction of thousands of dwellings and workshops; and it appears to have been thought that the finely organized human frame could receive no detriment from the permanent absence of agencies, which the Divine Creator and Upholder of the world has employed to quicken and develope vigorous life. Dr. Brown, of Chatham, has ably illustrated this in a late number of this Review. I also, in my essay on "Colliers' Diseases", in the same Journal (vol. ii), have already spoken at large of the injurious effects of the privation of the sun's rays on a large class of our artizans. I shall, therefore, now add but little on the subject. The most carefully collected statistics prove incontestably that, *cœteris paribus*, the inhabitants of underground and ill-lighted rooms are more frequently the victims of epidemic disease, than those persons who are better off in that *sole* point. Dr. Edward Smith, in his interesting paper recently read before the Royal Society, tells us that, in the course of his experiments on respiration, he found darkness the first and most powerful agent in decreasing the quantity of air breathed in a given time. *Ergo*, the absence of light *directly* tends to lower the vital forces, to check and render torpid the secreting and excreting processes, and to predispose the frame to the inroad of zymotic disease. This is not, however, generally credited. On the contrary, when a poor person inhabiting a dark, close hovel, is laid up with fever or scrofula, nobody thinks of attributing his illness to the right causes,—a want of God's light and pure air. No; it is a "chill", or over work; and the victim lies almost unheeded; and the epidemic gains ground, whilst the evils, which effectually preclude the usefulness of ordinary remedies, are perpetuated in ignorance.

Especially does *cholera* show great liking for darkness and humidity, its almost invariable associate. Perhaps, indeed, I am justified in saying, that no single predisposing cause, operates more certainly, than dwelling in a dark damp habitation. In July 1849, a severe outbreak of the epidemic occurred at a place called Woodhead, on the Sheffield line of railway, many miles distant from the nearest spot where the disease was raging. This was afterwards considered, and I think logically, attributable to the dark, damp tunnel, wherein the navigators were at that time working.

The connexion of physical with moral darkness, vice and immorality, and its consequent *in*direct but certain influence on the predisposition to disease, is also sufficiently patent. It was an awful truth, which was ages since enunciated from the lips of One who could not err,—"Every one that doeth evil hateth the light; neither cometh to the light, lest his deeds should be reproved."

---

I shall now proceed with the *second* class of the every-day causes of epidemics; and commence with the greatest and most extensive, viz.—

### I.—INTEMPERANCE.

No predisposing cause of epidemic disease, arising from the social habits of the people, has been rendered to observers more plainly apparent, than the habitual indulgence to excess in intoxicating drinks. In every town and district where fever or cholera makes its appearance, it is always seen that the drunkard is the first affected. I forbear to adduce examples, for I should not know where or which to select. The contents of my note-books would enable me readily to fill an entire number of this REVIEW, with proofs of the direct and potent agency of intemperance in the development and aggravation of epidemic diseases,—cholera especially. Fellow-labourers in the field, of higher note and better authority, are entirely confirmatory of my views and experience on this head. Dr. Elliotson, speaking of the visitation in 1832, remarks, "I clearly ascertained that those who had everything calculated to keep them in good health, but who indulged in spirit drinking, were sure to become victims." Dr. Babington, in his report, read last year before the Epidemiological Society, of the Visitation of Cholera in the Black Sea fleet, in 1854, gives among the very first predisposing causes—intemperance and bad food; and states that the immunity enjoyed by certain of the ships, depended on the sober and regular habits of the men. During the visitation of 1849, when I was busily engaged in the study and treatment of this awful epidemic, I noticed that the weekly extra indulgences, too commonly the habit among the working classes, on Saturday night and Sunday, almost invariably brought me a fresh and disproportionate number of fatal cholera cases on the Monday. The same significant circumstance was observed at Newcastle, in 1853. (See the *Lancet* for that year.) The Registrar's report for the Monday was, during each visitation, swelled weekly to a lamentable number, from the same obvious and preventible cause.

Reasoning *à priori* would lead us to expect this. The effect of the imbibition of alcohol is to diminish the amount of carbonic acid eliminated from the skin and lungs. Valentin, Prout, Fyfe, and Vierordt testify to this fact. Dr. E. Smith, in his admirable paper already alluded to, spoke of brandy and beer as greatly decreasing the respiration, and the quantity of carbonic acid exhaled. Dr. Bocker found, from his experiments on his own person, that these beverages diminished, by at least one-fifth, the amount of carbonic acid exhaled. In 1854, I performed the experiment of collecting the carbonic acid evolved from the lungs of two healthy individuals during one hour; both *before* and *after* administering a dose of alcohol, in the shape of whisky. In the first case, the quantity of gas evolved *previously* to taking the alcohol, was twelve hundred cubic inches; *after* it, nine hundred and fifty only.

In the case of the other person, the quantities were respectively nine hundred and six hundred and twenty cubic inches. These facts show that the presence of alcohol in the circulating current is always associated with a diminished percentage of carbonic acid in the air expired, and in the exhalation from the cutaneous surface. The blood becomes thereby loaded with effete carbon. I analysed, in 1850, the blood of two *delirium tremens* patients. It was, in both instances, deteriorated and deficient in plastic material. The proportion of fatty matters* was, in one case, eighty-six in one thousand parts; in the other, sixty-two. The healthy proportion is, certainly, not higher than ten. Lecanu found in the blood of a sot, the still higher proportion of one hundred and seventeen parts in one thousand. To apply the teachings of these results to the present subject; physiological science and experience alike instruct us, that the state of body the most favourable to the invasion and development of a zymotic aëreal poison, is set up by the presence in the blood of organic matter in a condition of change, of decomposition,—of fermentation, to speak familiarly. This state is readily induced by any agent, introduced from without, which checks the depuration of the blood in the lungs; and retains within it the effete products of its circulation. It is, *par excellence*, the condition of the blood of a drunkard. I shall not attempt here to discuss the much vexed question, as to whether the blood of a moderate habitual drinker is, *pro tanto*, in a similar state. I have no hesitation, however, in urging the following fact, which has received overwhelming proof, that the *least* habitual excess *beyond* a very moderate indulgence in fermented beverages, lowers the vital properties of the blood; destroys the normal tone of the nervous centres; and, as a constant *sequitur*, most powerfully predisposes the frame to the absorption of epidemic virus of whatever kind. Pure aërated blood affords the best safeguard against the attack of any epidemic. But the more perfect system of house ventilation, cleanliness, etc.. will fail to secure this, if, by the constant imbibition of alcohol in excess, the functions of the lungs and skin are interfered with, their healthy relations destroyed, and their waste products retained within the current.

To conclude on this point; experience has quite convinced me, that no other single social cause in operation is more active, or takes a more widely extended share in the diffusion and fatal aggravation of epidemic disease, than intemperance.

---

* These fatty compounds are formed from the waste carbon.

### II. FILTH.

What are the courts and back yards of houses in most low neighbourhoods, from the Scottish Highlands to the Land's End, but *parterres* of poison—hotbeds, sown thickly with the seeds of epidemic disease, and abundantly manured for the production of rich and perennial crops? When heavy rain falls on these accumulations of refuse after a long drought, and rapid evaporation by the heat of the sun afterwards takes place, a virus is engendered, which contaminates the surrounding air, spreads far and wide, and secures the fatal harvest of disease and suffering. Personal uncleanliness, owing to its relation with the important functions of the skin, has also much to do with the development of epidemic disease—especially of fevers. It depends, doubtless, in part on the facility of procuring the means of ablution; and might, therefore, with propriety have been discussed under the topic of water-supply. In fact, we find, in reviewing the evils arising from the social habits of the people, that these are in some measure dependant on the external circumstances surrounding them; and that the whole chain of such evils hangs closely together. Thus filth of person and habitation, improvidence, recklessness, and, to some extent, drunkenness and unchastity, are the almost inevitable consequences of bad drainage, foul water, dark and damp hovels. We may, however, draw a consolatory deduction from this truth, viz., that, by *removing* such outward circumstances, we may reasonably hope also to *destroy* the evil habits to which they have given birth.

### III. NEGLECT OF INFANTILE LIFE.

Carelessness, and the most improper management of infantile life, is a terrible and prominent characteristic of the principal manufacturing districts of England; especially of Lancashire, Yorkshire, and Lincolnshire. In pursuance of the eternal law of Nature, wherever we find a very high ratio of mortality, there do we also discover a more than average fecundity of race. In many of the large clothing towns in the north, thousands of infants are yearly brought into the world seemingly for no other purpose than to perish soon after seeing the light, like premature and blighted buds. The impurity of the dwellings in which these innocents are born, and the vitiated air they never change, is doubtless partly chargeable with the appalling mortality which prevails among them. But I wish in this section especially to call attention to causes in operation, which I believe to be yet more efficient in the diffusion of disease and the destruction of life. The detestable practice, of mothers who labour daily in the cotton-factories, leaving their helpless infants (as soon as they are themselves sufficiently recovered from their lyings-in) in the charge of some wretched old crone, who undertakes, perhaps, to "tend" five or six, is very general. Its almost inevitable result is the common adoption of the odious and never-to-be-sufficiently-reprehended practice of dosing the forlorn innocents with "*quietness*"—opium in some form. This horrid custom has already been ably exposed by Dr. Lyon Playfair and others. This narcotism at a tender age, not only *directly* produces brain-disease, scrofula, and other fearful maladies, but also establishes a permanent condition of system, in which the natural power of resistance to the inroads of disease is reduced to its lowest ebb. Hence, among children so circumstanced, an epidemic is almost sure to commit great ravages. But the mischief is not limited even to this. Such of these victims of a detestable and inhuman system as survive childhood, possess, as a consequence, even in adult age, a radical vice of constitution. The result of the present state of things in factories—especially the system of female and infant employment—is the production of a race of beings gradually sinking in the scale of intellectual vigour, virtue, and physical strength. Let any one who deems this an exaggerated and prejudiced statement, visit the manufacturing districts, and judge for himself. Let him enter the mills, and the cottages of the poor operatives; and not be dazzled by the glare of enormous wealth, the specious attraction of cotton-lords' mansions, and the triumphs of machinery. He will then discover what these triumphs have entailed upon us. Instead of benefiting by parental example, and exercising their bodies in the open air, the poor young children are sent at too early an age to the factories; where they have no moral restraint, where self-denial is unknown, and where they grow up slaves to the most degrading passions. In such a community, herded together, the empire of religion and reason gives place to the sway of brutality and sensualism. In fact, if the present condition of factory labour be not in some way amended, if some steps be not taken to rescue a great mass of our population from that abyss of vice, debasement and heathenism into which they are now hurrying, it seems clear, to my humble judgment, that civilization in the manufacturing north will speedily become but a synonyme for refinement of moral depravity; the grand ennobling truths of religion are there losing their hold on the public mind; their fruits are seldom met with; and (so far as regards the well-being and true enlightenment of the masses) the meridian of our national glory has reached its culminating point, and is already on the wane. In too many instances is a cotton-mill a "whited sepulchre"; outwardly showing evidence of wealth and power, but full of uncleanliness within.

In March and April, 1856, a severe outbreak of *measles* occurred in the district of Hindley (Lancashire). The ratio of mortality was very high, the number of fatal cases being one in four.* This declined and disappeared in May, and was succeeded by an epidemic of *hooping-cough* and *croup.* These were likewise of bad type. Of thirty-two cases of hooping-cough, five proved fatal, from the rapid supervention of pneumonia. Of seven cases of croup, three terminated fatally. Now I noticed (especially with regard to the measles) that nearly all the bad cases occurred among the children of factory operatives. Among other classes the disease was of a comparatively mild type, and ran its course favourably. My own children were early affected, and recovered without any aid from medicine. The worst cases were among the infants of weavers, brought up on *artificial food and opiates.*

### IV. UNHEALTHY OCCUPATIONS.

The "diseases of special occupations" have already attracted much attention. But there are many trades, not usually accounted unhealthy, but which, nevertheless, strongly predispose those persons engaged in them to epidemic disease, owing to the fact of their being carried on too frequently in unhealthy workshops. Tailors, compositors, milliners, etc. are often congregated together for many hours in stifling rooms, heated perhaps during the winter with stoves. Gas-light is justly deemed one of the great blessings and comforts of the present age, but it has its attendant evils, and those of great magnitude. It consumes the vital air much faster than any other artificial mode of illumination, and the products of its combustion are necessarily proportionably great. Even when the gas consumed is perfectly pure (and when is this the case?), the amount of carbonic acid generated speedily becomes sufficient to become highly deleterious, unless every care be taken to insure free ventilation. But it is generally much contaminated with sulphuretted hydrogen, which becomes sulphurous acid when burnt. This is very noxious to health, destroys the

---

* From a careful analysis of a very large number of cases, during various visitations, I estimate the average mortality as one in fourteen and a half.

appetite, and enfeebles the frame. At some future time I hope to publish some extended observations on the influence of diverse trades on the type of epidemic maladies.

I have now completed this brief sketch of a very important subject. My object has been to show, if possible, that there at present exist a large number of *remediable* causes of epidemic disease. It seems to me clear that *locality* has a far greater influence in inducing and spreading communicable disease than climate or season. There is much popular error on this point. An opinion is even now prevalent that cholera, influenza, scarlet-fever, typhus, etc. are mysterious scourges; "lighting where they list," and obeying no law but the will of an inscrutable Providence. This is a grave error. We have evidence enough to prove to the most sceptical that these fearful visitors are *not* indiscriminate. They affect and cling to the foul region, the overcrowded dwelling, the reeking sewer, the debauched and enfeebled frame. If it be asked, where do epidemics first break out, and where do they make most havoc? The answer, in accordance with an overwhelming amount of statistical evidence would be, "Almost always in dens of filth, and in low, dark and ill-ventilated places. For instance, where did the cholera first break out at St. Petersburgh in 1848? Among the boat population and the low parts adjacent to the river. Where at Dantzic? In mud barges. In Berlin, in 1832? Among the skippers in the boats. In Moscow? In a low, damp quarter, included within a bend of the river. In London, in 1849 and 1854? On the banks of the Thames and near the outlets of sewers. But let not the more affluent inhabitants of towns or rural districts console themselves with the idea that in their more favourable circumstances they are secure from danger. Once allow the demon of contagion to get a footing, and he will not, in the end, be confined to his natural element of filth and misery, but will overstep this boundary, and foray for victims among the families of the upper classes of society.

A word or two in the way of suggesting modes of remedying these bad influences, and I have done. The evils arising from want of ventilation might be partially met by a legislative enactment, insisting on the altitude of rooms in dwelling houses bearing a fixed proportion to their area, and prohibiting the letting of all new houses, until thoroughly inspected. Persons about to build houses should also bear the following sanitary points in mind. 1. *The situation to be dry.* 2. To be as elevated as possible compatibly with shelter. 3. *To be near a pure water supply.* 4. The houses not to be overshadowed by trees or taller buildings. But the greater part of the evil, especially that dependant on the social causes, can only be remedied by the gradual process of enlightenment among the people. This is to be effected by patient and quiet instruction. Clergymen might much enlarge their sphere of usefulness by descending occasionally from the pulpit, and lecturing on and enforcing sanitary matters. Medical men, on whom the public so much rely, should diligently teach and induce, by their example, others to teach, that *pure air is as necessary to health as good food;* that the north wind, striving to enter the crevices of a stifling room, is not an enemy to be shunned, but a friend to be welcomed; that a bedroom should always have a fire place in it, and an *un*stopped chimney; *that cesspool and fever are as cause and effect; that the use of foul water, sooner or later, induces disease;* that the blessed sunbeam is as necessary to the full vigour of man as of the plant; *that beer is not strength, nor can afford it;* that however well persons may be off, yet, if their bodies be enfeebled by the bestial vice of habitual intemperance, their prosperity is of no avail in the warding off disease; that the perspiratory tubing of a human being extends over twenty miles, and that a suspension of the functions of the skin is far more disastrous in its consequences than that of the bowels; that innocent children of tender age were not intended by the Creator to work for their bread in dreary rooms, reeking with steam and dust; but to gambol in the glad sunshine, and exercise their limbs in the open air. Finally, that disease is not so much flying about in the air, and hovering in unknown regions, as it is *developed in our homes, and fostered by evil habits.*

This is, *par excellence,* the age of progress, true and essential. And that which unmistakeably stamps this character upon it is the movement of Sanitary Reform. Its ultimate benefit lies far beyond its economy and immediate advantage, though these be very great. It prominently recognises the existence of sympathies and relations between all grades of social life, and that—

"One touch of Nature makes the whole world kin."

